# Taking a bite out of nutrition and arbovirus infection

**DOI:** 10.1371/journal.pntd.0006247

**Published:** 2018-03-29

**Authors:** James Weger-Lucarelli, Heidi Auerswald, Marco Vignuzzi, Phillipe Dussart, Erik A. Karlsson

**Affiliations:** 1 Viral Populations and Pathogenesis Unit, Institut Pasteur, Centre National de la Recherche Scientifique, Paris, France; 2 Virology Unit, Institut Pasteur du Cambodge, Institut Pasteur International Network, Phnom Penh, Cambodia; Oregon Health and Science University, UNITED STATES

## Abstract

Nutrition is a key factor in host–pathogen defense. Malnutrition can increase both host susceptibility and severity of infection through a number of pathways, and infection itself can promote nutritional deterioration and further susceptibility. Nutritional status can also strongly influence response to vaccination or therapeutic pharmaceuticals. Arthropod-borne viruses (arboviruses) have a long history of infecting humans, resulting in regular pandemics as well as an increasing frequency of autochthonous transmission. Interestingly, aside from host-related factors, nutrition could also play a role in the competence of vectors required for transmission of these viruses. Nutritional status of the host and vector could even influence viral evolution itself. Therefore, it is vital to understand the role of nutrition in the arbovirus lifecycle. This Review will focus on nutritional factors that could influence susceptibility and severity of infection in the host, response to prophylactic and therapeutic strategies, vector competence, and viral evolution.

## Author summary

As the old adage goes, you are what you eat. Proper nutrition is a cornerstone of health, and malnutrition can seriously impair the function of the immune system, resulting in increased infections or a more severe disease. Imbalanced or inadequate nutrition can also affect responses to vaccines or drugs that are vital for protection and treatment against viruses. A mosquito is also a product of what it eats. Nutrition during development and adult lifecycle can affect the feeding behavior of mosquitoes, thereby affecting transmission of viral diseases. Arthropod-borne viruses (arboviruses) are a major global health concern, especially in areas impacted by malnutrition. Understanding how nutrition can affect both humans and mosquitoes in the context of these viruses is vital to combating these diseases.

## Nutrition and infectious disease

Defined as any imbalance resulting in a deficiency or excess, malnutrition is the principal source of immunodeficiency worldwide [1]. Globally, as of 2014, it is estimated that 1.9 billion adults (>18 years of age) are overweight or obese by Body Mass Index (BMI)—18.5 kg/m^2^ to 24.9 kg/m^2^ = healthy weight, 25.0 kg/m^2^ to 29.9 kg/m^2^ = overweight, and ≥30 kg/m^2^ = obese—while 462 million are underweight. In children (<5 years of age), around 225 million are undernourished, around 42 million are overweight/obese [2, 3], and approximately 45% of deaths are linked to malnutrition, mainly in developing countries [3]. In lower- to middle-income countries, the rate of increase of childhood obesity is more than 30% higher than in developed countries. Greater than 65% of the global population lives in countries where overweight and obesity kill more people than underweight [2]. Undernutrition is also rampant throughout developed nations [4]. Overall, it is estimated that greater than one-third of the global disease burden could be eliminated by correcting malnutrition [5], and feeding children an adequate diet could prevent approximately 2.5 million deaths per year from pneumonia, diarrhea, malaria, and measles combined [6].

Malnutrition increases host susceptibility and severity of infection through several pathways, including weight loss, immune dysfunction, decreased epithelial integrity, and inflammation. In addition, infection itself can impact host nutritional status through infection-associated anorexia, altered metabolic rate, and altered dietary absorption, further complicating susceptibility and severity [1, 7, 8]. Indeed, frequency of exposure to infectious diseases increases the risk of poor nutrition in a vicious malnutrition–infection–malnutrition cycle [9, 10]. Overall, it is apparent that the interactions between nutrition and infectious disease are complex, with interplay between host, pathogen, and diet. This Review will discuss what is currently known (and unknown) about the relationship between nutritional status and arboviruses in both the vector and the human host.

## What is an arbovirus?

Arboviruses are spread to vertebrate hosts by hematophagous arthropod vectors. Transmission occurs via biological transfer, requiring successful replication in vector species as well as adequate viremia in the host before transmission is achievable. As of 1992, 535 virus species belonging to 14 virus families are registered in the International Catalog of Arboviruses [11], and new viruses are being described on a regular basis [12]. Of these species, greater than 100 are known to cause zoonotic diseases, mainly in four virus families: Togaviridae, Flaviviridae, Bunyaviridae, and Reoviridae [11]. While the majority of arboviruses circulate in tropical and subtropical regions, many arboviruses also have been introduced and thrive within temperate regions. Indeed, these viruses, along with their vector species, have spread exponentially in their geographical distributions in accordance with global trade routes and industrialization [13, 14]. This Review targets arboviruses transmitted by mosquitoes that have high public health importance and risk, namely chikungunya virus (CHIKV; Togaviridae), dengue virus (DENV; Flaviviridae), Zika virus (ZIKV; Flaviviridae), yellow fever virus (YFV; Flaviviridae), Japanese encephalitis virus (JEV; Flaviviridae), and West Nile virus (WNV; Flaviviridae). Combined, these viruses account for hundreds of millions of clinical/symptomatic infections globally, resulting in tens of thousands of deaths per year. However, symptoms in humans and animals range from mild to subclinical infection all the way to encephalitic or hemorrhagic, so the total number of cases per year may be underestimated (Table 1). In addition, due to the paucity of data on nutrition and arbovirus infection, other viruses of concern will also be mentioned where literature is available, including La Crosse virus (LACV; Bunyaviridae), Sindbis virus (SINV; Togaviridae), Ross River virus (RRV; Togaviridae), Western equine encephalitis virus (WEEV; Togaviridae), Rift Valley Fever virus (RVFV; Bunyaviridae), and St. Louis encephalitis virus (SLEV; Flaviviridae).

**Table 1:** Vectors, hosts, symptomology and estimated numbers of cases and deaths of selected arboviruses.

Virus	Family	Genus	Main vectors	Reservoir host	Characteristic symptoms (in clinical cases)	Cases/year (estimated)	Symptomatic or severe cases/year	Deaths/year	References
CHIKV	Togaviridae	*Alphavirus*	*Aedes* spp (in epidemic urban cycle: *A*. *aegypti*)	Primates	Fever, arthralgia, rash	Outbreak estimates only	Unknown	Unknown	[15]
DENV	Flaviviridae	*Flavivirus*	In enzootic cycle: arboreal *Aedes* spp. In epidemic urban cycle: *A*. *aegypti* and *A*. *albopictus*	Primates	Fever, hemorrhage	390 million (95% CI 284–528 million)	96 million (95% CI 67–136 million)	12,500 to 22,000	[16, 17]
ZIKV	Flaviviridae	*Flavivirus*	*Aedes* spp	Primates	Fever, rash	Outbreak estimates only	Unknown	Unknown	[18]
YFV	Flaviviridae	*Flavivirus*	*Aedes* and *Haemogogus* spp. (in urban cycle: *A*. *aegypt*i)	Primates	Hemorrhage, hepatitis	200,000	84,000 to 170,000 severe cases	29,000 to 60,000	[19, 20]
JEV	Flaviviridae	*Flavivirus*	*Culex* spp (especially *C*. *tritaeniorhynchus*)	Birds (Swine as secondary amplification host in epizootic cycle)	Fever, encephalitis	68,000	68,000 clinical cases	13,600 to 20,400	[21, 22]
WNV	Flaviviridae	*Flavivirus*	*Culex* species (especially *C*. *pipiens*)	Birds	Fever, encephalitis	Outbreak estimates only	30,000 to 50,000	10,000 to 15,000	[23, 24]

**Abbreviations:** DENV, dengue virus; CHIKV, chikungunya virus; JEV, Japanese encephalitis virus; WNV, West Nile virus; YFV, yellow fever virus; ZIKV, Zika virus.

## Review methodology

To review what is known on nutrition and arbovirus infection, a comprehensive search was conducted of the peer-reviewed literature available on Pubmed using a number of search terms. Combinations of terms for nutrition (nutrition, diet, feeding, obesity, body mass index, vitamin, micromineral) were used in combination with general and specific terms for arboviruses (arbovirus, alphavirus, flavivirus, bunyavirus, dengue, zika, chikungunya) and/or mosquito-associated terms (mosquito, *Culex*, *Aedes*, vector competence) to find papers related to the Review. All papers were included in the study as long as they pertained to nutritional influences on arboviruses.

## How can nutrition affect arbovirus infection, transmission and severity?

**Fig 1:**
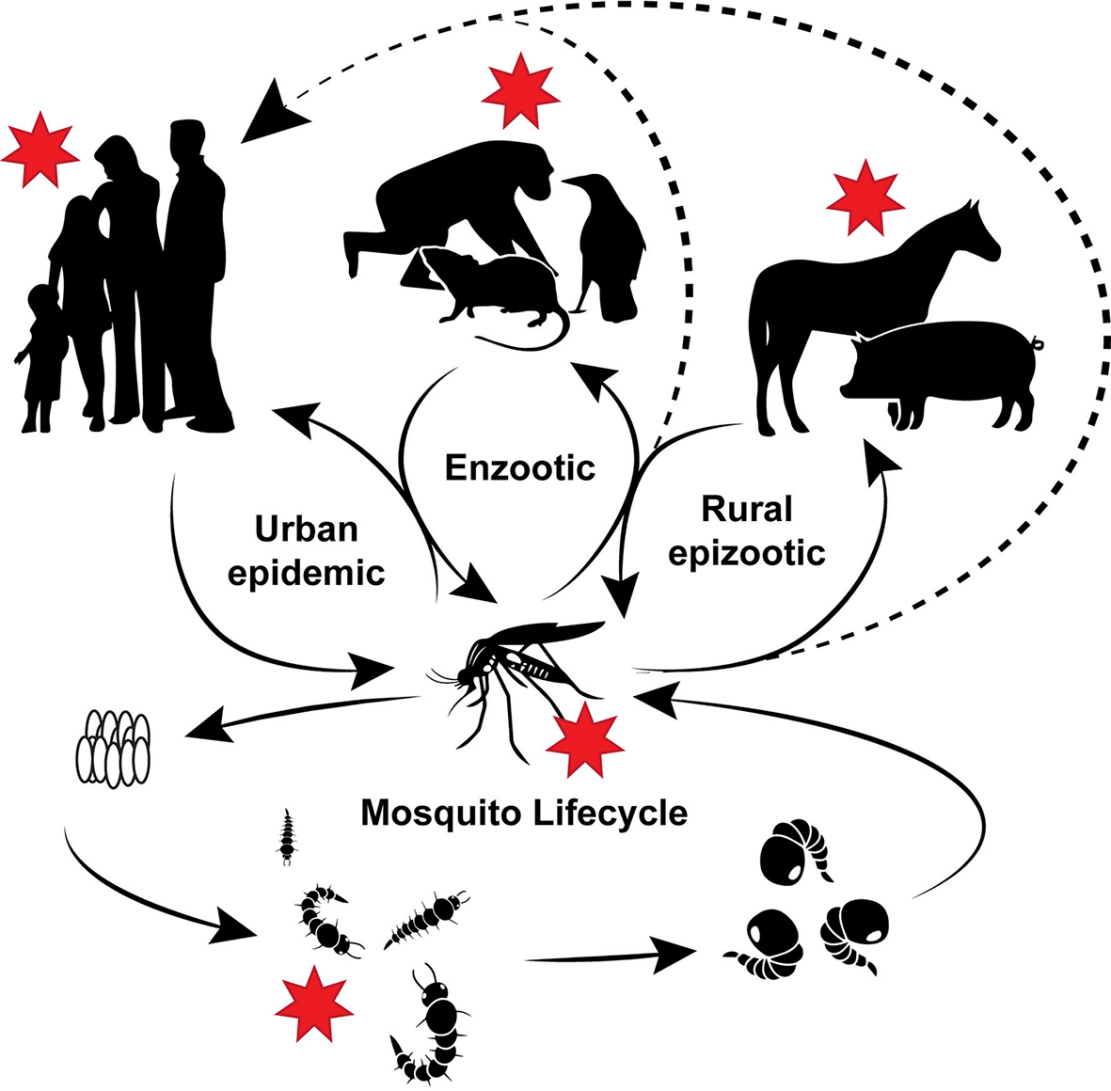
Influence of nutrition on the arbovirus vector–host cycle. Growth and development of mosquitoes as well as several pathways (epizootic, enzootic, and urban epidemic) could be impacted by the nutrition of both the host and the vector species. Red stars indicate areas where nutrition could have the most impact on susceptibility, severity of infection, and even vector competence.

The interplay of transmission cycle, host range, and evolution of arboviruses is a complex process. Arboviruses require a natural host as well as a vector for transmission [25]. While arthropod vectors abound, mosquitoes and ticks carry the most known virus species [11, 25, 26]. Further, of the 300 types of mosquitoes known to transmit arboviruses, female mosquitoes of the genera *Aedes* or *Culex* are most frequently associated with transmission [11, 25]. Arboviral diseases are generally associated with a specific vector and natural host species in rural epizootic and enzootic cycles. Humans and other large mammals tend to be accidental dead-end hosts for many of these cycles; however, spillover transmission to humans can lead to urban epidemic cycles where enzootic amplification is no longer required [25]. Since nutrition is essential for all organisms, numerous factors could be affected by changing nutritional status in reservoir and secondary amplification hosts as well as enzootic and/or endemic and epidemic vectors (Fig 1).

## Influence of nutrition on reservoir and secondary amplification hosts

**Fig 2:**
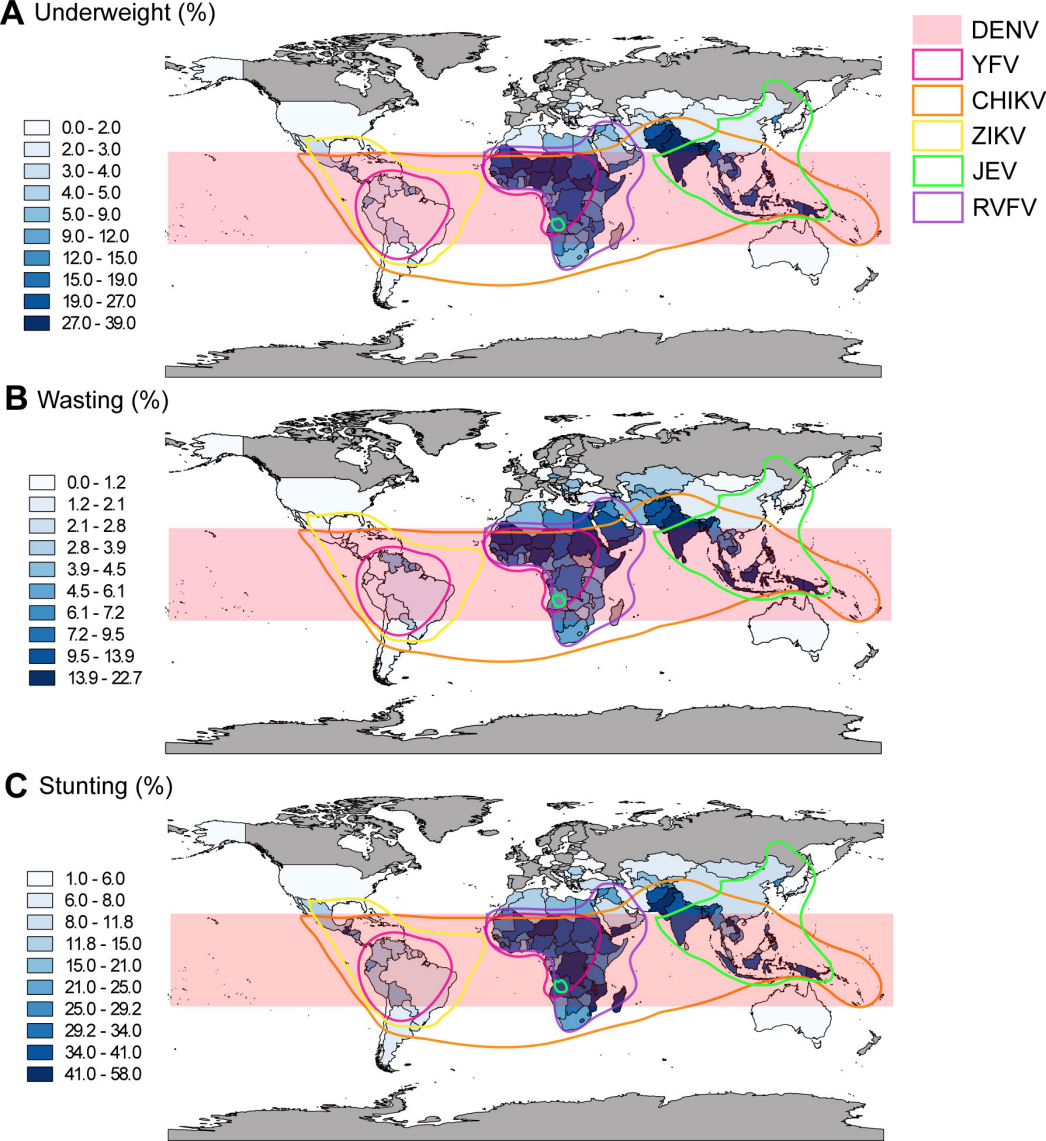
Correlation of malnutrition with reported distributions of arboviruses. Prevalence (by percent) of children under the age of 5 that are **(A)** underweight for their age, **(B)** wasted, or **(C)** stunted are shown in blue. Data are the most recent statistics for each country indicated available from the United Nations Children’s Fund (available at http://data.unicef.org) and were mapped using QGIS 2.18.12. Overlay colors indicate reported distributions of DENV (light red shading), YFV (pink border), CHIKV (orange border), ZIKV (yellow border), JEV (green border), and RVFV (purple border). Distributions are adapted from Weaver et al. 2017 [214]. CHIKV, chikungunya virus; DENV, dengue virus; JEV, Japanese encephalitis virus; RVFV, Rift Valley Fever virus; YFV, yellow fever virus; ZIKV, Zika virus.

Macronutrients and micronutrients are essential for a properly functioning immune system. Numerous nutritional states, such as undernutrition, obesity, and micronutrient deficiencies negatively impact immune function. These immune dysfunctions could then lead to alterations in host susceptibility or infection severity and possibly even increased transmission through changes in vector behavior. Given the global prevalence of malnutrition, particularly in areas hit hardest during arbovirus pandemics (Fig 2), it is essential to understand the connection between arbovirus and host and/or vector nutrition.

### Nutrition and host susceptibility to infection

Few prospective studies have been conducted on nutritional status and arbovirus susceptibility. Therefore, seroprevalence remains the primary means of associating nutrition, infection susceptibility, and arbovirus infection in human hosts (Table 2). Several studies show a strong association between high body weight and obesity and previous arboviral infection. In Madagascar, overweight pregnant women had significantly increased risk for CHIKV seroconversion [27]. On the island of La Réunion, overweight and obese individuals were also at increased risk during the 2006 outbreak [28, 29]. Obesity and increased body weight has also been associated with seropositivity for SINV in Sweden [30], DENV in Thailand [31], arboviruses of the genus *Phlebovirus* (family Bunyaviridae), and Toscana virus (TOSV; family Togaviridae) [32]. Overall, further prospective studies in arbovirus-endemic areas are crucial to define the relationship between infection susceptibility and nutritional status.

**Table 2:** Seroprevalence studies associating nutrition with infection susceptibility and arbovirus infection in humans.

Virus	Country	Age Range (years)	Study Design	Diagnostic Methods	Parameters of Malnutrition	*P*-value or odds ratio (OR)	References
CHIKV	Madagascar	12–50	Cross-sectional	IFA	Weight >70kg	OR: 9.75, *p* = 0.001.	[27]
CHIKV	La Réunion (France)	n/a	Case-control	ELISA	BMI >25 kg/m^2^	*p* < 0.0001	[29]
CHIKV	La Réunion (France)	Mean maternal age 28.6–29.1	Outbreak investigation	Unspecified serology RT-PCR	BMI >30 kg/m^2^	BMI = overweight, OR: 1.3. BMI = obese, OR: 1.6.	[28]
SINV	Sweden	25–74	Cross-sectional survey	EIA	BMI, waist circumference (cm) and diastolic blood pressure (mmHg)	BMI 26.8 versus 27.6, *p* = 0.2. Waist circumference 89.8 versus 93, *p* = 0.1. Diastolic blood pressure 79 versus 82, *p* = 0.037.	[30]
TOSV	Italy	4–75+	Cross-sectional	EIA	BMI >29.9 kg/m^2^	BMI = 25–29.9, OR 1.94. BMI >29.9, OR 2.73	[32]
DENV	Thailand	Mean age 5.8–9.7	Retrospective Cohort	ELISA and/or HI RT-PCR Virus isolation	Percent ideal body weight (IBW). Obesity defined as >110% IBW and malnutrition <75% IBW.	Malnourished: OR 0.48, *p* = 0.000. Lower chance of contracting dengue fever. Obese: OR 1.96, *p* = 0.000. Higher chance of contracting dengue fever.	[31]

**Abbreviations:** CHIKV, chikungunya virus; EIA, enzyme immunoassay; ELISA, enzyme-linked immunosorbent assay; HI, hemagglutination inhibition assay; IFA, immunoflouresence assay; n/a, not available; RT-PCR, real-time reverse transcriptase polymerase chain reaction; SINV, Sindbis virus; TOSV, Toscana virus.

In addition to body weight, the role of micronutrients on arbovirus infection susceptibility is understudied. Vitamins and minerals play a crucial role in immune function and are therefore essential to a proper antiviral defense. Vitamin D can reduce DENV infection and alter proinflammatory cytokine production in vitro [33, 34], and associations between vitamin D receptor gene polymorphisms and risk for DENV infection have been observed in host genetic studies [35]. Vitamin A levels (retinol and β-carotene) have been found to be decreased in DENV patients compared to healthy controls [36]. Zinc has also been shown to be an effective antiviral against many viruses [37]; however, little is known about its role in arbovirus infection aside from antiviral roles in vitro [38, 39]. Overall, further research is needed to scrutinize the relationship between micronutrient status and arbovirus susceptibility.

### Nutrition and arthropod host feeding risk: Can nutrition prevent being bitten?

Aside from host susceptibility, nutrition could also play a vital role in the ability and desire of mosquitoes to bite a given host. In fact, biting rate figures heavily into vectorial capacity, a measurement of the efficiency of vector-borne transmission [40]. Mosquitoes rely on olfaction for locating food sources. Several compounds commonly secreted in human skin, sweat, and breath, such as lactic acid and CO_2_, are potent mosquito attractants [26–28]. While host genetics plays a major factor in mosquito attractiveness [29], diet has also been suggested as a possible factor for altering individual body odors associated with attraction [41]. Indeed, before the scientific understanding of heritability of attraction, diet was (and possibly still is) the most cited cause of differential susceptibility to mosquito bites. Homeopathic and complementary medicine have suggested several bioactive dietary components that may prevent or encourage mosquito bites to augment traditional preventions and treatments; however, scientific evidence appears to be controversial.

Garlic has been touted as a mosquito repellent since before recorded history, possibly seeding the belief that garlic repels the vampiric behavior of blood consumption. In addition, garlic supplementation has long been used by dog and horse owners to prevent bites from blood-feeding insects. Scientifically, protection is suggested to be linked with the potent antimicrobial compound allicin [42]. While previous studies suggest some beneficial effect of garlic consumption, a more recent randomized, double-blind, placebo-controlled crossover study found no difference in bites or feeding behaviors of *A*. *aegypti* [43]. Consumption of vitamin B is also commonly prescribed for prevention of mosquito bites, especially vitamin B-1 (thiamine); however, no studies have shown any reduction in mosquito attraction with vitamin B supplementation [44]. Several other dietary ingredients have been purported to reduce mosquito attacks, such as onions, citrus fruits, lemongrass, chilies, apple cider vinegar, and vanilla. While compounds and/or essential oils found in these foods may prove to be effective mosquito repellants [45], no scientific literature is currently available on consumption of these foods in regards to reduction of mosquito attacks or feeding behavior.

Conversely, certain dietary components and nutritional states may increase host “attractiveness” and thereby increase bites. Similar to pregnant woman and individuals performing high-intensity exercise, obese and overweight individuals have increased CO_2_ production, increasing risk of mosquito bites [46]. Indeed, increased host body mass has been associated with increased and repeat feeding within groups of varied individuals [47]. Alcohol consumption may also alter susceptibility. Several studies have shown that consumption of alcohol as low as a single bottle of beer can increase host attractiveness to several mosquito species [48, 49]. Consumption of potassium-rich and salty foods increases lactic acid production, thereby increasing attractiveness. High-sugar foodstuffs could also increase attractiveness due to the need for nectars and/or plant sugars in the mosquito diet. These claims are currently scientifically unsubstantiated, and further work is necessary to define the role of host nutrition in attraction or prevention of mosquito bites [50].

### Nutrition and severity of infection in the host

Once the host has been bitten and become infected, infection severity is a significant factor in potential outcome. Compared to susceptibility, more studies have observed a relationship between disease severity and nutrition (Table 3). Obese individuals have an increased risk for inflammatory CHIKV sequelae [51], and diabetic status increases CHIKV severity and complications [52, 53]. Severity of WNV, including mortality, has also been associated with diabetes both during the initial outbreak of WNV in the Americas in 1999 [54] and later studies [55]. Furthermore, diabetic mice infected with WNV have increased mortality and impaired viral clearance as compared to healthy controls [56].

**Table 3:** Relationships between arboviral disease severity and nutritional factors in humans.

Virus	Age Range (years)	Study Design	Diagnostic Methods for Infection	Country	Parameters of Malnutrition	*p*-value or odds ratio (OR)	Conclusions	References
CHIKV	10–60+	Cohort	Confirmation by National Institute of Infectious Disease criteria	India	BMI <18.5 kg/m^2^ as underweight, 18.5–24.9 as normal, 25.0–29.9 as overweight, and ≥ 30 as obese	Overweight, OR: 1.3. Obese, OR: 2.07	High BMI is associated with CHIKV sequelae	[51]
CHIKV	20+	Case-control	Commercial rapid diagnostic testing	Haiti	Diabetes mellitus	Severe arthralgia, *p* = 0.0002. Days before arthralgia improvement, *p* < 0.0001. Days with fever, *p* = 0.0002	Diabetes associated with increased rate of myalgia, greater severity of arthralgia, and longer duration of fever compared to non-diabetic controls	[52]
CHIKV	16+	Case-control	Fever and/or polyarthralgia RT-PCR ELISA	La Réunion	Diabetes and ischemic heart disease	Diabetes, OR: 2.8. Ischemic heart disease, OR: 5.57	Patients hospitalized with CHIKV had higher rates of diabetes and ischemic heart disease compared to non-hospitalized controls	[53]
WNV	5–90	Outbreak surveillance	RT-PCR ELISA	USA	Diabetes mellitus	Encephalitis with muscle weakness, OR: 1.3. Death, OR: 5.1	Severe WNV disease associated with diabetes mellitus	[54]
WNV	0.4–95	Nested case-control	ELISA HI PRNT	USA	Diabetes mellitus	Symptomatic WNV infection, OR: 2.0. Death, OR: 3.5	Severe WNV disease associated with diabetes mellitus	[55]
DENV	2–15.9	Case-control	HI	Cuba	Defined as % P − E = A / B x 100. Where A = weight kg/height cm and B = 50th percentile of weight for age/ 50th percentile of height	*p* > 0.05	Did not find an association between nutritional status and dengue complications	[57]
DENV	0.3–15	Case-control	HI	Thailand	Nutritional status was determined using height, weight and mid-left arm circumference	No patients with 3rd degree malnutrition had severe dengue (no *p*-value could thus be reported)	Patients with severe malnutrition have reduced rates of severe dengue disease	[58]
DENV	0.5–1.5	Nested case-control	RT-PCR ELISA	Philippines	weight-for-age z-score as defined by WHO.	DHF versus other symptomatic dengue, *p* = 0.03	A WHO weight-for-age z score <−2 (i.e., undernutrition) during infancy was associated with low risk for DHF	[59]
DENV	<1	Cohort	ELISA	Vietnam	weight-for-age (WA), height-for-age (HA), weight-for-height (WH) z-score as defined by WHO	Developing DHF with undernutrition by WA or HA, *p* = 0.03 and *p* < 0.001, respectively) (negative association). Infants with undernutrition by WH developing DHF, *p* < 0.001 (positive association)	Infants with malnutrition as defined by WA or HA had reduced risk for developing DHF/DSS. Infants with malnutrition defined by WH had increased risk for DHF	[60]
DENV	Mean age 5.8–9.7	Retrospective cohort	ELISA and/or HI RT-PCR Virus isolation	Thailand	Percent ideal body weight (IBW). Obesity defined as >110% IBW and malnutrition <75% IBW	Malnourished versus control, risk of DSS, *p* = 0.004. Obese versus control, risk of DF/DHF, *p* <0.001	In patients with DHF, under or overnutrition was associated with severe disease or unusual clinical presentations. Undernutrition was associated with decreased risk of dengue infection	[31]
DENV	0.8–16	Retrospective cohort	Clinical diagnostic criteria defined by the WHO	Thailand	Body weight as a percentile of the normal range for the age	Not reported	The occurrence of severe DHF is more prominent in patients with body weight greater than 50th percentile for age	[61]
DENV	0–14	Case-control	Clinical diagnostic criteria defined by the WHO	Thailand	Weight-for-height	Obesity versus Control, risk of DHF, *p* = 0.001, OR 3.00	Obesity is associated with development of DHF	[62].
DENV	5–12	Case-control	Clinical diagnostic criteria defined by the WHO ELISA	El Salvador	weight-for-age (WA), BMI-for-age z-score as defined by WHO	Malnourished versus control, risk of DHF, *p* = 0.09	No differences were observed related to nutritional status and development of dengue fever or hemorraghic fever as compared to controls	[63]

**Abbreviations:** CHIKV, chikungunya virus; DENV, dengue virus; DF, dengue fever; DHF, dengue hemorrhagic fever; ELISA, enzyme-linked immunosorbent assay; HA, height-for-age; HI, hemagglutination inhibition test; IBW, ideal body weight; PRNT, plaque reduction neutralization test; RT-PCR, real time polymerase chain reaction; WA, weight-for-age; WH, weight-for-height; WNV, West Nile virus

Perhaps most is known about the association between nutritional status and DENV severity. Early observational studies suggested no association between poor nutrition and DENV hemorrhagic disease in Thailand [64]. However, later reports showed that malnourished children experience less severe cases of DENV versus those that are well nourished [65, 66]. Further reports provided evidence for these anecdotes [57, 58], and subsequently, this association has also been observed in children in the Philippines and Vietnam [59, 60]. Conversely, obesity has been associated with increased severity of dengue hemorrhagic fever and unusual disease presentation, such as encephalopathy and fluid overload, in several [31, 61, 62] (but not all [63]) studies. Unfortunately, many of these studies do not use consistent definitions for malnutrition or obesity and therefore can be difficult to compare. A large multinational study with consistent parameters for assessment of nutritional status is necessary to truly settle this debate.

Possible links between micronutrients and arbovirus disease severity have also been examined in several studies. Associations between vitamin D levels (measured by overall vitamin D status or vitamin D-binding protein) and outcome of dengue fever are mixed [67–69]. Other micronutrients such as zinc [70–72], vitamin A [36], iron [73, 74], copper [73], chromium [75], and vitamin E [76] have also been reported to be associated with development of severe DENV disease. High doses of intravenous vitamin C have been used to treat infection with CHIKV, although more work is needed for confirmation [77]. While more research must be done to improve our understanding of the role of micronutrients and arbovirus disease, there are promising results that suggest ameliorating these nutrient deficiencies or excesses may reduce disease burden and severity.

### Nutrition and prophylactic and therapeutic strategies in the host

Prophylactic and therapeutic strategies are crucial for preventing infection and mitigating disease severity. Nutrition can play a crucial role in these vital strategies. Several arbovirus vaccines are now available or currently in development [78–81] and are critical for many arbovirus-endemic areas of the world, many of which have high rates of one or more nutritional deficiencies (Fig 2). The live-attenuated YFV vaccine, 17D, is by far the most widely administered arbovirus vaccine and has the only vaccine study with nutritional components. Children with kwashiorkor (severe protein deficiency) had a significantly lower seroconversion rate to 17D (12.5%) versus healthy controls (83.3%) [82]. To date, no studies have looked at arboviral vaccines in the obese host; however, several studies have shown obesity can reduce vaccine response or vaccine effectiveness against other pathogens [83–85]. Micronutrients, especially vitamins A and D, are also crucial for vaccination [86–89]. Unfortunately, there is a paucity of information on micronutrients and arbovirus vaccine response. One study showed vitamin A deficiency did not reduce response to YFV 17D vaccine [90]. However, individuals deficient in vitamin A had reduced lymphocyte and cytokine proliferation following vaccination, which could affect long-term vaccine efficacy. Further work is necessary on these crucial nutrients, especially since these viruses are endemic in areas of the world with significant micronutrient deficiencies [3].

In addition to vaccination, the use of antivirals as a therapeutic strategy against arboviruses is critical. During the 1873 YFV epidemic in Memphis, Tennessee, iced champagne was recommended as a curative [91] with negligible effect. Despite research efforts, antiviral treatment for arboviruses has not significantly progressed since that time. While a few antiviral compounds have been tested, few have shown success outside of small-animal laboratory models, and no specific antivirals are currently available for arboviruses [92–94]. Due to the lack of antivirals currently available, several groups have been investigating natural products and medicinal plants as a resource for combating these viruses. These products have a long history as part of traditional medicines and diets [95, 96]. While not necessarily predictive of actual consumption, in vitro studies have revealed potential antiviral effects associated with several common food items. Curcumin, a principal component of the turmeric root, inhibits cell binding of DENV, ZIKV, and CHIKV [97, 98]. Polyphenols such as delphinidin (found in cranberries, grapes, and pomegranates) and epigallocatechin gallate (found in green tea and bananas) have been investigated for their strong antiviral effects against WNV, ZIKV, DENV, and CHIKV in vitro [99, 100]. Papaya leaf and garlic extracts alter the immune response during dengue infection, presumably reducing symptoms during infection without directly affecting viral replication [101, 102]. Additional studies should be performed to assess the antiviral efficacy of these plant-derived compounds. Another issue to consider is the effect of malnutrition on pharmacokinetic processes, drug responses, and toxicity. Diet and nutrition are extremely important to the pharmatoxicological properties of chemicals and malnutrition has been shown to generate therapeutic inadequacies and changes in drug toxicity [103–105].

## Influence of nutrition on arboviral vectors

Aside from the reservoir and secondary hosts, nutrition is also vital for the growth and development of arbovirus vectors and can affect numerous pathways associated with vector susceptibility, viral load and burden, willingness to feed, and even antivector control. For the purposes of this Review, we will focus on nutritional influences on the mosquito genera *Aedes* and *Culex*. The life cycle of hematophagous mosquitoes incorporates four main stages: eggs, larvae (subdivided into four stages called instars), pupae, and adults (Fig 1). Only larvae and adult mosquitoes feed during their development, whereas the other two are inactive stages of metamorphosis in which development is dependent on nutritional reserves [106, 107].

Larvae live in aquatic habitats, feed on organic detritus, bacteria, algae, protozoa, and other microorganisms [108, 109], and seem to feed randomly depending only on abundance. Carbohydrates, minerals (especially calcium), protein (at least the amino acids glycine, leucine, isoleucine, histidine, arginine, lysine, tryptophan, threonine, and methionine), vitamin B complex (thiamin, riboflavin, pyridoxine, nicotinic acid, pantothenic acid, and folic acid), and fat (cholesterol, lecithin, or the fat components of yeast) [110–116] are essential for mosquito development. Appropriate nutrition supports the development from larvae to pupa stage as well as formation of reserves for adult mosquitoes. These reserves consist mainly of lipids and glycogen and support the survival of adults [117–119]. Feeding habits of adult mosquitoes are gender-dependent. Male mosquitoes feed exclusively on sugar and live for around ten days. Female mosquitoes (like some *Aedes* and *Culex* species) consume sugar as well but need blood for the development of their ovaries and eggs (anautogenous). Both sugar and blood are used to produce glycogen and fat reserves in females, which are necessary for their lengthened life span (40–60 days) and egg production [120–122].

Bacteria and microbiome also appear to be vitally important to mosquito development and behavior. All bacteria found in larvae or adult mosquitoes have also been isolated in the water used for oviposition, indicating that larvae ingest the bacteria and transfer them to the adult stage [123, 124]. Therefore, adults and especially females might take up bacteria from the oviposition sites simply by water contact [125]. Female *C*. *pipiens* are more attracted to oviposition habitats where *Klebsiella* and *Aeromonas* bacteria are present. These bacteria not only act as food source but are also proposed to be symbionts that can be transferred vertically to the next stages and even the next generation [124–127].

### Nutrition and vector feeding and/or host-seeking behavior

Changes in larval nutritional status can directly affect adult mosquitoes and therefore arbovirus infection in the vector or host. Insufficient diet or starvation during larval development leads to smaller, often weaker adult mosquitoes with fewer reserves and a shorter life span, thereby decreasing chances of transmission and/or infection [128–131]. Restricted larval diet can affect the sex ratio (more males versus females) of *C*. *molestus*, resulting in fewer mosquitoes searching for a blood meal [132]. Larval nutrition also has a potential effect on adult host-seeking behavior. *A*. *aegypti* females originating from nutrition-deprived larvae are smaller and show less host-seeking behavior [131]. Interestingly, smaller *Aedes* females also probe more often and take multiple blood meals during one gonotrophic cycle (life cycle of feeding and laying of eggs) [118, 133–136]. This increased contact could enhance the potential of single-host transmission by smaller females despite seeking a host less frequently. On the other hand, larger females have increased host-seeking behavior to cover higher energy requirements [137, 138], increased survival, and more reserves [139], resulting in extended flights [134, 140] and thereby increasing the possibility for transmission to multiple hosts.

Adult nutrition can also impact potential transmission. Feeding on sugar (carbohydrates) prolongs the life span of mosquitoes [119, 141–143]. Indeed, a nutrient-rich adult diet can compensate for life-shortening effects of nutrient deprivation during larval stages in *Aedes* [144–146] and *Culex* [147] species. Sugar deprivation leads to starvation and death [141, 143, 148–152]. Sugar-seeking behavior can also affect propensity of the vector to seek a blood meal. Generally, sugar feeding inhibits the search for a vertebrate host [120, 145, 153–155]. *C*. *restuans* females feed on nectar when they are unfed (not blood-fed) and when they are carrying eggs (gravid), whereas *A*. *vexans* females take nectar only while unfed (not blood-fed and/or not gravid). However, both species rarely feed on sugar while digesting a blood meal [156]. Interestingly, females of *C*. *nigripalpus* show enhanced host-seeking behavior following sucrose feeding, while starved females preferentially feed on honey [157]. For *A*. *aegypti* females, field observations show infrequent consumption of sugar [158]; however, regular sugar intake is observed under laboratory conditions leading to higher fecundity [121]. In carbohydrate-deprived *A*. *aegypti*, gravid females attempt to obtain blood meals more often [159].

In contrast to sugar consumption, protein components of blood, specifically amino acids, are necessary for development of the ovarian follicles and oviposition [109]. As such, the host species greatly influences egg number and, subsequently, number of mosquitoes able to transmit arboviruses. *C*. *quinquefasciatus* females show a higher fertility and fecundity when fed with chicken blood compared to bovine [160]. *A*. *aegypti* females preferentially feed on humans but will produce more eggs if they feed from other animals, possibly due to the low isoleucine content in human blood [161, 162]. Indeed, *A*. *aegypti* have adapted to feeding on protein-rich, isoleucine-poor human blood by taking additional blood meals [118, 133, 138, 163–166]. The importance of amino acids for initiation of egg development has been demonstrated by several feeding experiments utilizing artificial diets. *A*. *aegypti* fed a meal containing only 12 amino acids, including isoleucine, were able to produce eggs [167]. Similarly, feeding of isoleucine-rich globulins versus isoleucine-poor human hemoglobin induced the development of the ovaries and eggs [168, 169]. Interestingly, arbovirus infection itself can also alter feeding behavior of mosquitoes under laboratory conditions. *A*. *aegypti* females infected with DENV feed longer [170] and LACV infected *A*. *triseriatus* females probed more but took less amount of blood than uninfected mosquitoes [171].

### Nutrition and vector competence

Nutrition could also affect vector competence itself (Table 4). For the purposes of this Review, vector competence describes the ability of the vector to become infected with an arbovirus and to show potential to transmit the virus to a host. Overall, existing data on nutritional impacts on vector competence are limited and extremely controversial. Several studies have found that smaller adult females raised from nutrient-deprived larvae showed an increased vector competence. The most extensive studies have been performed using *A*. *triseriatus* and LACV. Smaller females originating from nutrient-deprived larvae had significantly higher viral titers and increased oral transmission versus normal-sized (control) or large (overfed) mosquitoes [172, 173], possibly through higher dissemination rates within the mosquito itself [174]. Inverse correlation between mosquito size and vector competence has been further confirmed with LACV in field-caught *A*. *triseriatus*, DENV and SINV in *A*. *albopictus*, and DENV and RRV in *A*. *aegypti* [175–178]. Similar results are observed with *Culex* mosquitoes. *C*. *tritaeniorhynchus* reared with a low nutrient diet as larvae had higher JEV titers [179], and smaller females are slightly more susceptible to WNV infection [180].

**Table 4:** Studies observing the effect of nutrition on vector competence.

Effect of smaller size	Putative cause of decreased size	Mosquito Species	Virus	Findings in smaller females	References
Increased vector competence	Decreased food quantity during larval development	*A*. *triseriatus*	LACV	Increased oral transmission rates and higher dissemination rates	[173, 174]

	Decreased food quantity during larval development	*A*. *albopictus*	DENV	Increased susceptibility	[181]
* *	Decreased food quantity during larval development	*A*. *aegypti*	RRV	Larger blood meals (including higher amount of virus uptake) relative to body size	[176]
* *	Lower quality food	*C*. *tritaeniorhynchus*	JEV	Increased dissemination rates	[179]
* *	Decreased food quantity during larval development and increased larvae density	*C*. *tritaeniorhynchus*	WNV	Higher infection rates	[180]
* *	Decreased food quantity (and other factors) during larval development	*A*. *triseriatus* (field-caught)	LACV	Increased dissemination and transmission	[175]
No effect on vector competence* *	Decreased food quantity	*A*. *vigilax*	RRV	No difference	[182]
* *	Decreased food quantity	*C*. *tarsalis*	WNV	No difference	[183]
* *	Decreased food quantity	*C*. *annulisostris*	Murray Valley encephalitis virus	No difference	[184]
* *	Altered salt content in natural habitat	*C*. *tarsalis*	WEEV, SLEV	No difference	[185]
Reduced vector competence	Decreased food quantity and increased density during larval development	*A*. *aegypti*	DENV	Lower infection rates	[186]
* *	Decreased food quantity during larval development	*A*. *aegypti*	RRV	Lower infection rates	[176]

**Abbreviations:** DENV, dengue virus; LACV, La Crosse virus; JEV, Japanese encephalitis virus; RRV, Ross River virus; SLEV, St. Louis encephalitis virus; WEEV, Western equine encephalitis virus; WNV, West Nile virus.

Other studies have shown opposite or no effect of mosquito size on vector competence. Larger mosquitoes have been shown to be more susceptible to arbovirus infection, particularly *A*. *aegypti* and DENV [186] and *A*. *albopictus* and CHIKV [187], possibly due to increased viral receptors in the gut [176]. Some studies have shown no correlation between mosquito size and vector competence [185]. Overall, these studies do suggest that nutrition during larval stages can affect vector competence of the adult mosquito; however, further work is necessary to elucidate the exact mechanism associated with these changes.

Aside from mosquito size, mosquito microbiome may also play an important role in vector competence. Elimination of endogenous bacteria in *A*. *aegypti* mosquitoes increases susceptibility to DENV [188], and probiotic transfer of *Proteus* bacteria into the midgut increases resistance. Mechanistically, microbiomes may protect mosquitoes from certain arbovirus infections by production of secondary antiviral metabolites [189]. In contrast, microbiota can also decrease the expression of immune genes and therefore increase the susceptibility [188].

### Nutrition as a means of vector control

Prevention of host infection is highly dependent on effective vector control. Most strategies aim to kill larvae directly, interfering with development or sterilizing the adults. Most commonly, these efforts are achieved with chemical growth regulators [190]. However, these hormone analogs can also affect benevolent insect species, and resistance is already found worldwide [191, 192]. Therefore, there is an urgent need for novel vector control strategies, such as nutritional components. As stated above, the mosquito microbiome is necessary for reproduction and can influence vector competence. The most popular vector control strategy utilizing bacteria is based on endosymbiotic *Wolbachia*. *Wolbachia* are not ingested directly but are maternally transmitted from infected females to their offspring. Introduction of new *Wolbachia* species into field populations reduces the mosquito reproduction as well as infection susceptibility and transmission potential for several arboviruses such as DENV [193–196], CHIKV [193–197], and YFV [197]. The mechanism is not completely understood; however, direct competition between the endosymbiotic bacteria and arbovirus is postulated [195, 196, 198]. Specific *Wolbachia* strains can also decrease mosquito lifespan, reducing the likelihood of arbovirus transmission [199].

Another strategy for vector control is to introduce larvicidal components that are ingested by larvae in situ. Several bacteria produce larvicidal proteins that have been successfully applied for vector control [200, 201], and several plant extracts and leaf litter also demonstrate larvicidal activity [202, 203]. Algae ingested by mosquito larvae can also have larvicidal effects [204], mainly through production of toxins [205] or starvation [126, 206, 207]. More work is necessary to identify other dietary components or interventions that are more effective in panspecies mosquito population reduction.

## Influence of nutrition on arboviruses themselves

RNA viruses, such as arboviruses, intrinsically exist as heterogeneous, highly mutable populations that can quickly take advantage of environmental conditions [208]. It is by this mechanism that arboviruses can quickly adapt to new vectors and hosts [209]. Since nutritional status has such a profound influence on the host and/or vector, it can also act as a driving force in the emergence of new viral variants [210]. Nutritional status has been found to directly influence virulence in several RNA viruses, including coxsackievirus [211] and influenza virus [212, 213]. Overall, changes in nutritional status can result in point mutations, increasing virulence and/or adaptation when reintroduced to a new host. These mutations could result from reduced viral population bottlenecks due to compromised immune responses or novel viral mutations from increased exposure to inflammation and reactive oxygen species [210]. While no studies have directly observed the influence of nutrition on arbovirus mutation and population dynamics, future work will focus on these factors in different nutritional states in both host and vector species.

## Conclusions and future perspectives

The number of arbovirus infections increases steadily on a yearly basis and the exact causes for the increased frequency of arboviral outbreaks are not fully understood. Combining the global prevalence of malnutrition with continual arbovirus pandemics, increasing frequency of autochthonous transmission, and the paucity of adequate vaccination and antiviral strategies, it is essential to understand the connection between arbovirus susceptibility and severity and host and vector nutrition. Nutritional status is known to play a major role in immune status and in the development, physiology, and behavior of several mosquito species. Taken together, modulation of nutritional status or amelioration of malnutrition seems to be a targetable method of interrupting transmission as well as reducing susceptibility and disease severity.

**Key learning points**

- Nutrition is an understudied aspect of arbovirus infections.
- Compromised nutritional status (malnutrition) is rampant in areas with emerging or endemic arbovirus infections.
- Nutrition is a critical component of host susceptibility to infection and disease severity, with malnutrition leading to increased chance of becoming infected or having more severe infection outcomes.
- Malnutrition can severely impact ability to respond to prophylactic and therapeutic strategies against arbovirus infection.
- Nutritional status of vector species, especially during larval development, can significantly impact host-seeking behaviors and vector competence, resulting in changes in virus transmission.

**Top five papers**

1. Kalayanarooj S, Nimmannitya S. Is dengue severity related to nutritional status? Southeast Asian Southeast Asian Journal of Tropical Medicine and Public Health. 2005;36(2):378–84.
2. Ahmed S, Finkelstein JL, Stewart AM, Kenneth J, Polhemus ME, Endy TP, et al. Micronutrients and Dengue. American Journal of Tropical Medicne and Hygeine. 2014;91(5):1049–56.
3. Klowden MJ, Blackmer JL, Chambers GM. Effects of larval nutrition on the host-seeking behavior of adult Aedes aegypti mosquitoes. Journal of the American Mosquito Control Association. 1988;4(1):73–5.
4. Grimstad PR, Walker ED. Aedes triseriatus (Diptera: Culicidae) and La Crosse virus. IV. Nutritional deprivation of larvae affects the adult barriers to infection and transmission. Journal of Medical Entomology. 1991;28(3):378–86.
5. Lefèvre T, Gouagna L-C, Dabiré KR, Elguero E, Fontenille D, Renaud F, et al. Beer Consumption Increases Human Attractiveness to Malaria Mosquitoes. PLoS ONE. 2010;5(3):e9546. doi: 10.1371/journal.pone.0009546
